# Dietary whole sweet sorghum silage alters jejunal morphology, selected cecal bacterial taxa, and meat quality in heavy finishing pigs

**DOI:** 10.3389/fmicb.2026.1971504

**Published:** 2026-10-14

**Authors:** Shaoyang Shi, Jiandong Wei, Yonggang Zhang, Xuejun Yuan, Xianglan Shang, Xiao Li, Huajie Zhou, Yanping Jiang, Weiren Yang, Min Zhou, Shuzhen Jiang

**Affiliations:** 1 Key Laboratory of Efficient Utilization of Non-Grain Feed Resources (Co-construction by Ministry and Province), Ministry of Agriculture and Rural Affairs, College of Animal Science and Technology, Shandong Agricultural University, Tai’an, China; 2 College of Biosystems Engineering and Food Science, Zhejiang University, Hangzhou, China; 3 Qingdao Huanshan Biotechnology Co., Ltd., Qingdao, China; 4 College of Life Sciences, Shandong Agricultural University, Tai’an, China; 5 Jiangji Town Animal Husbandry Station, Ningyang, Shandong, China; 6 Zhongcheng Feed Technology Co., Ltd., Feicheng, China; 7 College of Agricultural Science and Technology, Shandong Agriculture and Engineering University, Zibo, China

**Keywords:** cecal microbiota, dietary fiber, growth performance, heavy finishing pigs, jejunal morphology, meat quality, whole sweet sorghum silage

## Abstract

Whole sweet sorghum silage (WSS) is a locally available forage resource with potential application in sustainable swine production, yet its biological effects on the intestinal environment and production-related traits of heavy finishing pigs (defined here as pigs entering the trial at approximately 132 kg BW) remain unclear. This study evaluated the effects of replacing 20 percentage units of corn with WSS (on an as-fed basis) on growth performance, meat quality, jejunal morphology, and cecal bacterial composition. Seventy-two Duroc × Landrace × Yorkshire pigs were fed the corn-based control diet (CON) or the WSS diet for 30 d. Compared with CON, WSS reduced average daily gain and feed intake and increased the feed-to-gain ratio. It also decreased backfat thickness while increasing loin-eye area and marbling score. WSS reduced 48-h drip loss and increased inosine monophosphate concentrations in both the longissimus dorsi and psoas major muscles. Jejunal villus height and the villus-height-to-crypt-depth ratio were increased in WSS-fed pigs. No significant differences were detected in alpha-diversity indices or unweighted UniFrac community structure between diets, whereas an exploratory linear discriminant analysis effect size procedure identified *Streptosporangium*, *Kribbella*, *Oxalobacter*, and *Planifilum* as taxa associated with WSS and *Bacteroides*-related features as associated with CON. Because the diets were not nutrient-matched, these responses reflect the complete dietary contrast. Overall, WSS was associated with a pattern of reduced growth performance accompanied by selected changes in carcass and meat-quality traits, concurrent jejunal morphological adaptation, and exploratory cecal bacterial signals that warrant mechanistic validation.

## 1 Introduction

Corn is the predominant energy source in swine diets owing to its high starch digestibility, consistent nutritional value, and excellent palatability (Woyengo et al., 2014; Tan et al., 2021). However, increasing competition for corn among livestock production, human consumption, and industrial utilization has stimulated interest in locally available alternative feed resources (Popp et al., 2016). Consequently, identifying alternative feed sources that are locally available and suitable for partial replacement of corn has become an important strategy for improving the sustainability and resilience of pig production.

Sweet sorghum is a high-yielding C_4_ crop characterized by rapid biomass accumulation, a high soluble-sugar concentration, and strong tolerance to drought and salinity (Qu et al., 2014; Wu et al., 2010; Li et al., 2013; Umakanth et al., 2019). Unlike sorghum grain, whole-plant sweet sorghum provides both fermentable carbohydrates and structural fiber, making it a potential roughage source for heavy finishing pigs. Moreover, ensiling allows year-round use while improving storage stability and partially modifying cell-wall carbohydrates through microbial fermentation (Xu et al., 2022b; Zhang et al., 2015; Bai et al., 2023). Consequently, whole sweet sorghum silage (WSS) differs markedly from corn in moisture, starch, and fiber composition. These differences are particularly relevant to pigs because structural carbohydrates escaping digestion in the upper gastrointestinal tract provide substrates for microbial fermentation in the hindgut.

Dietary fiber is an important determinant of the intestinal microbial ecosystem in pigs. Changes in fiber source and concentration can alter substrate availability for microbial fermentation and thereby affect the relative abundance and metabolic potential of the intestinal microbiota (Pu et al., 2022; Luo et al., 2022). Such responses are often accompanied by changes in intestinal morphology, nutrient utilization, and host physiology. Importantly, microbial responses to dietary fiber are not uniform but depend on fiber physicochemical properties, inclusion level, dietary context, and the physiological stage of the animal. The late finishing period is also important for carcass composition and the development of pork-quality characteristics. Emerging evidence suggests that diet-induced changes in the intestinal environment may occur concurrently with alterations in host metabolism and skeletal muscle characteristics. This has led to increasing interest in diet–microbiota–host interactions and the potential gut–muscle axis in livestock. Nevertheless, whether the intestinal and microbial responses to WSS are accompanied by changes in carcass and meat characteristics remains poorly understood. Establishing these associations is an important first step toward determining whether WSS exerts biological effects beyond its contribution to dietary nutrient supply.

Therefore, this study evaluated the effects of partially replacing corn with 20% WSS (on an as-fed basis) in complete diets for heavy finishing pigs on growth performance, carcass characteristics, meat quality, jejunal morphology, and cecal microbial composition. We hypothesized that dietary WSS would modify intestinal morphology and selected cecal bacterial relative-abundance patterns because of its greater fiber content and that these responses would occur concurrently with changes in animal performance and meat quality. Given the growing recognition of the gut–muscle axis in livestock, such concurrent responses may provide a rationale for future mechanistic studies; however, the present experiment was not designed to test microbial or intestinal mediation of muscle characteristics or meat quality.

## 2 Materials and methods

### 2.1 Experimental design and animal management

Seventy-two Duroc × Landrace × Yorkshire finishing pigs with an initial body weight (BW) of 131.6 ± 2.13 kg, comprising equal numbers of barrows and gilts, were allocated to 12 pens, with six pigs per pen (three barrows and three gilts). Pens were randomly assigned to either a corn-based control (CON) or whole sweet sorghum silage (WSS) treatment, with six pens per treatment. The CON group was offered the basal diet, whereas the WSS group received a diet in which WSS replaced 20 percentage units of dietary corn. Following a 7-d adaptation period, pigs were fed the experimental diets for 30 d, with unrestricted access to feed and water throughout the trial. The diets were formulated according to the nutrient requirements recommended by the <ref> National Research Council, (2012)</ref>, and their ingredient composition and analyzed nutrient levels are presented in Table 1. The facilities were cleaned and disinfected before the experiment. Recorded ambient temperature ranged from 18 to 22 °C, and relative humidity was approximately 65%.

**TABLE 1:** Ingredient composition (%, as-fed basis) and nutrient levels of the experimental diets.

Ingredient	CON	WSS^3^	Nutrient^2^	CON	WSS
whole sweet sorghum silage	0.00	20.00	DE, MJ/kg	13.97	12.85
corn	60.00	40.00	Crude protein	15.46	14.38
soybean meal (43% CP)	16.00	16.00	Crude fiber	3.86	6.81
wheat bran	12.00	12.00	Calcium	0.65	0.64
wheat secondary flour	9.00	9.00	Total phosphorus	0.58	0.52
soybean oil	0.70	0.70	Available phosphorus	0.25	0.24
CaHPO_4_	0.60	0.60	Lysine	0.75	0.72
pulverized limestone	1.20	1.20	Methionine	0.25	0.22
NaCl	0.40	0.40	Sulfur-containing amino acids	0.51	0.48
premix^1^	0.10	0.10	Threonine	0.54	0.50
Total	100.00	100.00	Tryptophan	0.17	0.16

^1^Supplied per kg of diet: vitamin A, 1440 international units (IU); vitamin D_3_, 150 IU; vitamin E, 11.8 IU; vitamin K_3_, 0.51 mg; vitamin B_1_, 0.97 mg; vitamin B_2_, 2.4 mg; pantothenic acid, 7.62 mg; nicotinic acid, 29.89 mg; pyridoxine, 1.39 mg; biotin, 0.05 mg; folic acid, 0.3 mg; vitamin B_12_, 0.01 mg; Mn (as MnSO_4_⋅H_2_O), 2.5 mg; Fe (as FeSO_4_⋅H_2_O), 40.5 mg; Zn (as ZnSO_4_⋅H_2_O), 51.4 mg; Cu (as CuSO_4_⋅5H_2_O), 3.06 mg; I (as KIO_3_), 0.14 mg; Se (as Na_2_SeO_3_), 0.20 mg.

^2^DE, digestible energy; CP, crude protein. Nutrient levels were calculated from the original formulation matrix and are expressed on an as-fed basis.

^3^CON was the corn-based control diet. WSS was the complete experimental diet containing 20% whole sweet sorghum silage on an as-fed basis.

All procedures involving animals complied with the Guide for the Care and Use of Laboratory Animals and were approved by the Animal Care and Use Committee of Shandong Agricultural University (SDAUA-2020-0710; approval date: 10 July 2020).

### 2.2 Preparation of whole sweet sorghum silage

Fresh whole-plant sweet sorghum bearing intact panicles was harvested 115–130 d after sowing at a stubble height of approximately 40 cm and chopped to a length of 2–3 cm. The material was mixed with a silage inoculant containing *Lactiplantibacillus plantarum* ( > 1 × 10^9^ colony-forming units/mL), *Bacillus* spp. ( > 1 × 10^9^ colony-forming units/mL), cellulolytic bacteria ( > 1 × 10^9^ colony-forming units/mL), and cellulase (1 × 10^9^ U/mL), compacted, and wrapped in bales weighing 1000 ± 5 kg. The bales were stored outdoors in the shade at approximately 15–25 °C for 60 d. Fresh material assigned to each of the six separately wrapped bales was sampled immediately before wrapping, and the corresponding silage was sampled from the same bale after 60 d of ensiling. Unique bale identifiers established six paired bale-level observations. Air-dried whole sweet sorghum (WS) and WSS samples were analyzed for dry matter (DM), crude protein (CP), ether extract (EE), neutral detergent fiber (NDF), and ash, and the results are presented in Table 2. The proximate components were determined using the corresponding AOAC International procedures (AOAC International, 1995). During the feeding trial, exposed silage surfaces were resealed after removal, inspected daily for visible mold and heating, and deteriorated material was not fed.

**TABLE 2:** Chemical composition of sweet sorghum before and after ensiling.

Item^1^	WS	WSS	*p*-value
DM^2^	28.50 ± 0.21	27.80 ± 0.16	0.065
CP	8.30 ± 0.15	7.80 ± 0.08	0.084
EE	2.80 ± 0.06	4.50 ± 0.06	< 0.001
NDF	59.00 ± 0.29	48.10 ± 0.26	< 0.001
Ash	6.37 ± 0.08	6.12 ± 0.08	0.048

^1^WS, whole sweet sorghum; WSS, whole sweet sorghum silage; DM, dry matter; CP, crude protein; EE, ether extract determined by Soxhlet extraction; NDF, neutral detergent fiber. Values are means ± standard error of the mean (SEM) from six paired bales (*n* = 6 pairs). Technical replicate measurements were averaged within each sample before analysis, and pre- and post-ensiling values from the same bale were compared as described in section “2.8 Statistical analysis.”

^2^The DM value represents the actual fresh dry matter content of the crops at harvest or opening; all other chemical components are expressed on a 100% dry matter basis.

### 2.3 Sample collection

Representative diet samples were collected before the feeding trial and stored in sealed containers under cool, dry conditions until chemical analysis. The BW of each pig was recorded on days 0 and 30, and the mean BW of the six pigs in each pen was calculated at each time point. Pen-level average daily gain (ADG) was calculated as the change in pen mean BW from days 0 to 30 divided by 30 d. Feed disappearance was recorded daily for each pen; average daily feed intake (ADFI) was calculated at the pen level, and feed-to-gain ratio (F/G) was calculated for each pen as ADFI/ADG.

At the completion of the 30-d feeding trial, pigs were fasted for 12 h before sampling. Before slaughter, three pens within each treatment were designated to provide a barrow and three to provide a gilt. Within the designated sex in each pen, the pig whose final BW was closest to the pen mean BW was selected. Thus, the six slaughter pigs in each treatment comprised three barrows and three gilts, with one pig sampled from each independent pen. The selected pigs were electrically stunned and subsequently euthanized by exsanguination.

All carcass measurements and muscle, jejunal, and cecal samples were obtained from the same selected pig in each pen (six pigs per treatment). Following slaughter, longissimus dorsi and psoas major muscle samples were collected for the assessment of meat quality, proximate composition, inosine monophosphate (IMP), and total hydrolyzed amino acid composition. For intestinal histology, an approximately 2-cm section of the mid-jejunum was immediately excised, gently rinsed with physiological saline, and fixed in Bouin’s solution for histological analysis. Approximately 2 mL of cecal digesta was aseptically transferred into sterile ribonuclease- and deoxyribonuclease-free cryogenic tubes, rapidly frozen in liquid nitrogen, and stored at −80 °C until microbial analysis.

### 2.4 Histological analysis

Mid-jejunal samples were immersed in Bouin’s fixative, processed through graded dehydration, embedded in paraffin, and cut into 5-μm sections. Following hematoxylin and eosin staining, intestinal morphology was evaluated under a light microscope (BX41, Olympus, Tokyo, Japan). Three nonconsecutive sections were prepared per animal. Villus height (VH) and crypt depth (CD) were measured using ImageJ software (National Institutes of Health, Bethesda, MD, USA), and the villus-height-to-crypt-depth ratio (VH/CD) was calculated. At least ten well-oriented villi and their corresponding crypts were measured per sample. All morphometric measurements were performed by an investigator blinded to dietary treatment.

### 2.5 Meat quality analysis

Muscle pH was measured in the longissimus dorsi and psoas major at 45 min and 24 h after slaughter with a portable waterproof pH meter (HI 99163, Hanna Instruments, Carrollton, TX, USA). Surface color was characterized by lightness (L*), redness (a*), and yellowness (b*) values using a CR-410 colorimeter (Konica Minolta, Mississauga, ON, Canada) with D65 illumination, with three readings obtained for each sample. For drip loss, samples were suspended at 4 °C for 48 h, and drip loss was expressed as a percentage of the initial sample weight (Honikel, 1998). Cooking loss was expressed as the proportion of weight lost after the samples were heated in a 75 °C water bath for 30 min and then cooled to room temperature (Honikel, 1998). Meat tenderness was evaluated as Warner–Bratzler shear force perpendicular to the orientation of the muscle fibers using a C-LM3B tenderness meter (Beijing Tianxiang Feiyu Instrument Equipment Co., Ltd., Beijing, China) (Honikel, 1998). After 24 h of carcass chilling at 4 °C, marbling was visually assessed on the exposed loin surface between the 10th and 12th ribs according to National Pork Producers Council standards (National Pork Producers Council, 1999), and the mean of the scores assigned independently by three trained evaluators who were blinded to dietary treatment was used for analysis. Backfat thickness and loin-eye dimensions were measured at the 12th rib, and loin-eye area (cm^2^) was calculated as loin-eye height × loin-eye width × 0.7.

### 2.6 Proximate composition, inosine monophosphate, and amino acid analysis

Muscle samples were freeze-dried, ground, and analyzed for moisture, EE, CP, and total hydrolyzed amino acids according to AOAC International methods (AOAC International, 1995). EE was determined by Soxhlet extraction, CP by the Kjeldahl method, and amino acids after acid hydrolysis using an automatic amino acid analyzer. Table 3 presents concentrations of selected amino acids, expressed as g/100 g DM, rather than a complete amino acid profile. IMP was extracted with perchloric acid and quantified by reverse-phase high-performance liquid chromatography with ultraviolet detection at 254 nm. Results were expressed as mg/g fresh muscle.

**TABLE 3:** Muscle inosine monophosphate and selected total hydrolyzed amino acid concentrations in finishing pigs fed the CON or WSS diet.

Item	CON	WSS^1^	*p*-value
Longissimus dorsi muscle			
Inosine monophosphate, mg/g fresh muscle	2.51 ± 0.02	2.74 ± 0.03	< 0.001
Glutamate	12.29 ± 0.13	13.30 ± 0.13	< 0.001
Aspartic acid	7.21 ± 0.14	8.28 ± 0.13	< 0.001
Phenylalanine	3.48 ± 0.11	3.45 ± 0.10	0.808
Tyrosine	2.06 ± 0.06	2.46 ± 0.09	0.004
Alanine	5.53 ± 0.12	5.65 ± 0.07	0.370
Glycine	3.55 ± 0.09	3.54 ± 0.09	0.905
Psoas major muscle			
Inosine monophosphate, mg/g fresh muscle	1.90 ± 0.03	2.10 ± 0.03	0.001
Glutamate	10.84 ± 0.15	11.75 ± 0.12	< 0.001
Aspartic acid	5.30 ± 0.10	6.42 ± 0.10	< 0.001
Phenylalanine	2.97 ± 0.07	3.39 ± 0.13	0.002
Tyrosine	1.37 ± 0.06	1.97 ± 0.04	< 0.001
Alanine	4.00 ± 0.49	4.47 ± 0.10	0.367
Glycine	2.58 ± 0.08	2.62 ± 0.09	0.743

^1^CON was the corn-based control diet. WSS was the complete diet containing 20% whole sweet sorghum silage on an as-fed basis, included at the expense of 20 percentage units of corn. Values are means ± SEM from one pig sampled from each of six independent pens per treatment (*n* = 6 pigs per treatment). IMP is expressed as mg/g fresh muscle. Amino acids are expressed as g/100 g DM and represent total hydrolyzed amino acids released from muscle protein, not free taste-active amino acids.

### 2.7 Cecal microbiota analysis

Microbial genomic deoxyribonucleic acid (DNA) was isolated from cecal digesta using a commercial extraction kit (Omega Bio-Tek, Inc., Norcross, GA, USA) following the manufacturer’s protocol. The bacterial 16S ribosomal RNA (rRNA) gene was amplified targeting the V3–V4 hypervariable region with the primer pair 341F (5′-CCTAYGGGRBGCASCAG-3′) and 806R (5′-GGACTACNNGGGTATCTAAT-3′). Amplicon libraries were subsequently sequenced on an Illumina NovaSeq 6000 (Novogene Bioinformatics Technology Co., Ltd., Beijing, China) using a 250-bp paired-end strategy. After sequencing, paired-end reads were assembled with Fast Length Adjustment of Short reads (FLASH) and subjected to quality control using fastp. Potential chimeric sequences were detected and removed with VSEARCH. The resulting high-quality sequences were clustered into operational taxonomic units (OTUs) at a 97% sequence similarity threshold using UPARSE v7.0.1001. Taxonomic annotation of representative OTU sequences was performed with the Mothur algorithm against the SILVA 138.1 reference database. The OTU abundance table was normalized to 41,476 sequences per sample for alpha-diversity and UniFrac analyses. Alpha diversity, including the Chao1, abundance-based coverage estimator (ACE), Shannon, and Simpson indices, was calculated using QIIME v1.9.1. Per-sample sequencing yields and counts retained at each processing stage are provided in Appendix Table A1. Beta diversity was visualized by principal component analysis (PCA) and unweighted UniFrac-based principal coordinates analysis (PCoA) in Quantitative Insights Into Microbial Ecology (QIIME) v1.9.1. Community structure based on unweighted UniFrac distances was tested by permutational multivariate analysis of variance (PERMANOVA) with 999 permutations. Differential taxa were identified by linear discriminant analysis effect size (LEfSe), using a Wilcoxon rank-sum significance criterion of *p* < 0.05 and an absolute linear discriminant analysis (LDA) score threshold of > 3.5. No multiplicity adjustment was applied, and the LEfSe results were interpreted as exploratory.

### 2.8 Statistical analysis

Statistical analyses were conducted using IBM SPSS Statistics 22.0 (IBM Corp., Armonk, NY, USA). The pen was the experimental unit for dietary treatment and all growth-performance variables; therefore, ADG, ADFI, and F/G were analyzed using six independent pen observations per treatment. For slaughter, carcass, meat-quality, histological, and microbiota outcomes, the same selected pig from each of the six independent pens supplied the observation (*n* = 6 pigs per treatment). Technical replicates were averaged within each experimental unit before analysis. The Shapiro–Wilk test was used to evaluate the normality of data distribution. Differences between dietary treatments were analyzed using independent-samples *t*-tests, and pre- and post-ensiling composition was analyzed using paired-samples *t*-tests with the bale as the paired experimental unit. Data are presented as means ± standard error of the mean (SEM), and *p* < 0.05 was considered significant. Microbiota-specific analyses are described in section “2.7 Cecal microbiota analysis.”

## 3 Results

### 3.1 Chemical composition of whole sweet sorghum before and after ensiling

As shown in Table 2, ensiling increased ether extract (EE) and decreased neutral detergent fiber (NDF) and ash concentrations when expressed on a dry matter basis (*p* < 0.05). In contrast, the actual dry matter (DM) content of the fresh material and the crude protein (CP) concentration expressed on a dry matter basis did not differ before and after ensiling (*p* > 0.05).

### 3.2 Growth performance

In this study, neither initial nor final body weight (BW) differed between treatments (*p* > 0.05). Relative to the corn-based control diet (CON), the WSS diet significantly reduced average daily gain (ADG) and average daily feed intake (ADFI) and increased the feed-to-gain ratio (F/G) (*p* < 0.05, Table 4).

**TABLE 4:** Growth performance of finishing pigs fed the CON or WSS diet.

Item	CON	WSS^2^	*p*-value
Initial body weight, kg	131.62 ± 4.20	131.58 ± 1.52	0.994
Final body weight, kg	159.19 ± 4.24	155.53 ± 1.73	0.443
Average daily gain, kg/d	0.92 ± 0.01	0.80 ± 0.01	< 0.001
Average daily feed intake, kg/d	3.08 ± 0.04	2.91 ± 0.03	0.008
F/G^1^	3.34 ± 0.02	3.64 ± 0.08	0.003

^1^F/G, feed-to-gain ratio; ADFI, average daily feed intake; ADG, average daily gain. ADG was calculated from the change in pen mean BW from days 0 to 30. ADFI and F/G were calculated for each pen from feed disappearance converted to an air-dry basis; F/G is expressed as kg air-dry feed/kg BW gain.

^2^CON was the corn-based control diet. WSS was the complete diet containing 20% whole sweet sorghum silage on an as-fed basis, included at the expense of 20 percentage units of corn. Values are means ± SEM based on six pens per treatment (*n* = 6).

### 3.3 Carcass traits

As shown in Table 5, dietary inclusion of WSS significantly decreased backfat thickness and increased loin-eye area and marbling score compared with the CON diet (*p* < 0.05).

**TABLE 5:** Carcass traits of finishing pigs fed the CON or WSS diet.

Item	CON	WSS^1^	*p*-value
Backfat thickness, mm	18.33 ± 0.49	16.17 ± 0.31	0.004
Loin-eye area, cm^2^	59.72 ± 2.15	65.54 ± 1.26	0.042
Marbling score, 1–10	2.33 ± 0.33	3.67 ± 0.21	0.007

^1^CON was the corn-based control diet. WSS was the complete diet containing 20% whole sweet sorghum silage on an as-fed basis, included at the expense of 20 percentage units of corn. Values are means ± SEM from one pig sampled from each of six independent pens per treatment (*n* = 6 pigs per treatment).

### 3.4 Muscle proximate composition

In the psoas major muscle, the WSS diet significantly increased CP (*p* < 0.05), whereas DM and EE did not differ between treatments (*p* > 0.05; Table 6). In the psoas major muscle, the WSS diet significantly increased CP, whereas DM and EE were unchanged (*p* < 0.05, Table 6).

**TABLE 6:** Muscle proximate composition of finishing pigs fed the CON or WSS diet.

Item	CON	WSS^1^	*p*-value
Longissimus dorsi muscle, %			
Dry matter	27.43 ± 0.29	27.46 ± 0.15	0.941
Crude protein	20.18 ± 0.45	20.73 ± 0.44	0.382
Ether extract	4.83 ± 0.10	4.04 ± 0.13	< 0.001
Psoas major muscle, %			
Dry matter	26.20 ± 0.11	25.65 ± 0.31	0.108
Crude protein	17.95 ± 0.30	18.84 ± 0.10	0.012
Ether extract	5.52 ± 0.29	4.73 ± 0.34	0.092

^1^CON was the corn-based control diet. WSS was the complete diet containing 20% whole sweet sorghum silage on an as-fed basis, included at the expense of 20 percentage units of corn. Values are means ± SEM from one pig sampled from each of six independent pens per treatment (*n* = 6 pigs per treatment).

### 3.5 Meat quality

The effects of WSS on meat quality are shown in Tables 7, 8. In the longissimus dorsi muscle, the WSS diet significantly increased pH and redness (a*) at 24 h and decreased drip loss at 48 h (*p* < 0.05), whereas pH at 45 min, lightness (L*) and yellowness (b*) at both measurement times, redness (a*) at 45 min, cooking loss, and shear force did not differ between treatments (*p* > 0.05). In the psoas major muscle, the WSS diet significantly decreased yellowness (b*) at 45 min and drip loss at 48 h (*p* < 0.05), whereas the remaining meat-quality traits were unchanged (*p* > 0.05).

**TABLE 7:** Meat-quality traits of the *longissimus dorsi* muscle in finishing pigs fed the CON or WSS diet.

Item	CON	WSS^1^	*p*-value
Longissimus dorsi muscle			
pH at 45 min	6.29 ± 0.14	6.23 ± 0.13	0.760
pH at 24 h	5.71 ± 0.03	5.83 ± 0.03	0.011
Lightness (L*) at 45 min	41.76 ± 0.52	40.48 ± 0.49	0.103
Redness (a*) at 45 min	8.44 ± 0.39	8.98 ± 0.25	0.268
Yellowness (b*) at 45 min	9.56 ± 0.16	9.23 ± 0.20	0.241
Lightness (L*) at 24 h	44.19 ± 0.96	44.60 ± 0.87	0.758
Redness (a*) at 24 h	10.87 ± 0.18	11.64 ± 0.09	0.003
Yellowness (b*) at 24 h	11.90 ± 0.26	11.41 ± 0.13	0.126
Drip loss at 48 h, %	7.00 ± 1.00	3.00 ± 0.10	< 0.001
Cooking loss, %	35.00 ± 2.00	33.00 ± 1.00	0.557
Shear force, *N*	82.17 ± 1.27	79.47 ± 2.79	0.399

^1^ CON was the corn-based control diet. WSS was the complete diet containing 20% whole sweet sorghum silage on an as-fed basis, included at the expense of 20 percentage units of corn. Values are means ± SEM from one pig sampled from each of six independent pens per treatment (*n* = 6 pigs per treatment).

**TABLE 8:** Meat-quality traits of the psoas major muscle in finishing pigs fed the CON or WSS diet.

Item	CON	WSS^1^	*p*-value
Psoas major muscle			
pH at 45 min	6.12 ± 0.09	6.11 ± 0.03	0.872
pH at 24 h	5.74 ± 0.05	5.78 ± 0.02	0.432
Lightness (L*) at 45 min	37.83 ± 0.08	37.87 ± 0.59	0.957
Redness (a*) at 45 min	17.11 ± 0.41	18.09 ± 0.42	0.131
Yellowness (b*) at 45 min	9.60 ± 0.24	8.65 ± 0.22	0.015
Lightness (L*) at 24 h	40.63 ± 0.90	41.27 ± 2.82	0.832
Redness (a*) at 24 h	17.59 ± 0.43	18.22 ± 0.22	0.226
Yellowness (b*) at 24 h	11.45 ± 0.13	11.08 ± 0.31	0.294
Drip loss at 48 h, %	5.00 ± 0.20	4.00 ± 0.30	0.016
Cooking loss, %	38.00 ± 1.00	37.00 ± 1.00	0.605
Shear force, N	87.56 ± 1.05	87.06 ± 2.05	0.830

^1^CON was the corn-based control diet. WSS was the complete diet containing 20% whole sweet sorghum silage on an as-fed basis, included at the expense of 20 percentage units of corn. Values are means ± SEM from one pig sampled from each of six independent pens per treatment (*n* = 6 pigs per treatment).

### 3.6 Muscle inosine monophosphate and total hydrolyzed amino acid composition

The WSS diet significantly increased IMP concentrations and the concentrations of glutamate, aspartic acid, and tyrosine measured after total hydrolysis in both muscles and additionally increased phenylalanine in the psoas major muscle (*p* < 0.05). Phenylalanine in the longissimus dorsi muscle and alanine and glycine in both muscles did not differ between treatments (*p* > 0.05, Table 3).

### 3.7 Jejunal morphology

Representative jejunal sections stained with hematoxylin and eosin showed preserved mucosal architecture in both treatments (Figure 1A). Villi in the CON sections (A1–D1) appeared shorter, whereas those in the WSS sections (E1–H1) appeared longer and retained an intact epithelium. Morphometric analysis showed that the WSS diet significantly increased villus height and the villus-height-to-crypt-depth ratio (VH/CD) (*p* < 0.01, Figure 1B), whereas crypt depth did not differ between treatments (*p* > 0.05).

**FIGURE 1:**
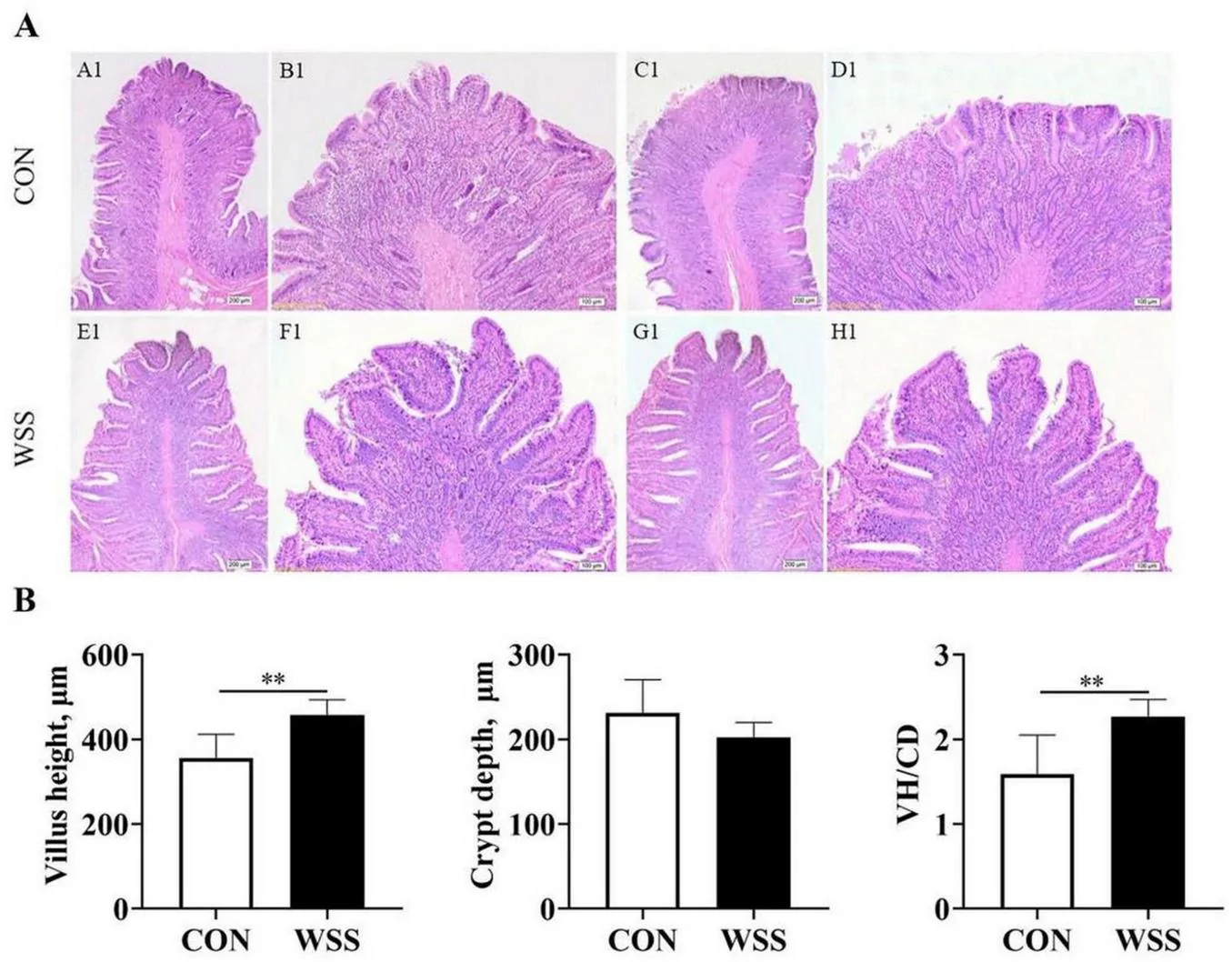
Jejunal histomorphology of finishing pigs fed the CON or WSS diet (one pig sampled from each of six independent pens per treatment; *n* = 6 pigs per treatment). **(A)** Representative hematoxylin and eosin (H&E)-stained jejunal sections from the CON (A1–D1) and WSS (E1–H1) groups. Scale bars indicate 200 μm in A1, C1, E1, and G1 and 100 μm in B1, D1, F1, and H1. **(B)** Quantitative analysis of villus height (left), crypt depth (middle), and the villus-height-to-crypt-depth ratio (VH/CD, right). CON denotes the corn-based control diet, and WSS denotes the complete diet containing 20% whole sweet sorghum silage on an as-fed basis. Values are means ± standard error of the mean (SEM) after subsample measurements were averaged within each animal. **p < 0.01 versus CON.

### 3.8 Cecal microbiota

Across the 12 samples, 968,435 raw read pairs were obtained, with 69,914–86,385 read pairs per sample (median, 82,282). After merging and quality filtering, 68,745–84,682 tags per sample were retained. Following chimera removal, 717,614 effective tags remained, corresponding to 50,278–64,224 per sample (median, 61,549; Appendix Table A1). A total of 2,407 operational taxonomic units (OTUs) were shared between treatments, whereas 1,958 and 2,318 OTUs were unique to CON and WSS, respectively (Figure 2A). Rarefaction curves continued to rise, although their slopes decreased at higher sequencing depths (Figure 2B). The species-accumulation plot also continued to increase across the 12 sampled animals (Figure 2C).

**FIGURE 2:**
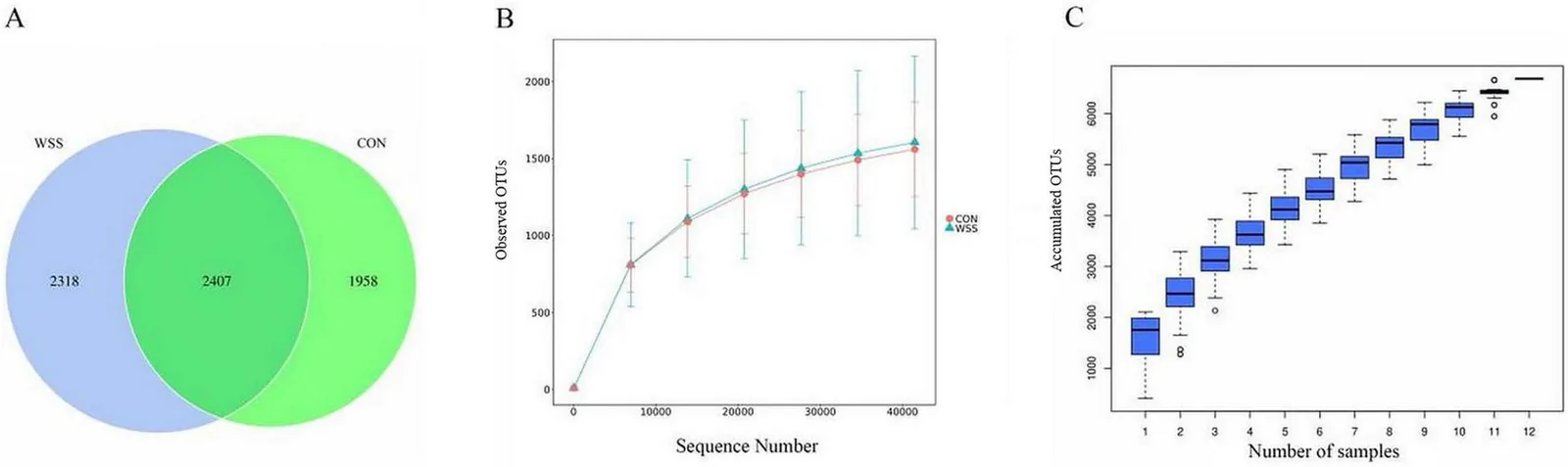
Cecal operational taxonomic unit (OTU) richness and sequencing coverage in finishing pigs fed the CON or WSS diet (one pig sampled from each of six independent pens per treatment; *n* = 6 pigs per treatment). **(A)** Shared and unique OTUs. **(B)** Rarefaction curves. **(C)** Species-accumulation box plot. CON denotes the corn-based control diet, and WSS denotes the complete diet containing 20% whole sweet sorghum silage on an as-fed basis, included at the expense of 20 percentage units of corn.

Bacterial richness (Chao1 and ACE indices) and diversity (Simpson and Shannon) did not differ between treatments (*p* > 0.05, Figures 3A–D). PCA and unweighted UniFrac-based PCoA showed overlapping group distributions (Figures 3E, F). Consistent with the ordinations, PERMANOVA based on unweighted UniFrac distances detected no significant treatment difference (999 permutations, *p* > 0.05). The archived analysis output did not retain an exact *p*-value, pseudo-F statistic, or R^2^, and these values were therefore not reconstructed.

**FIGURE 3:**
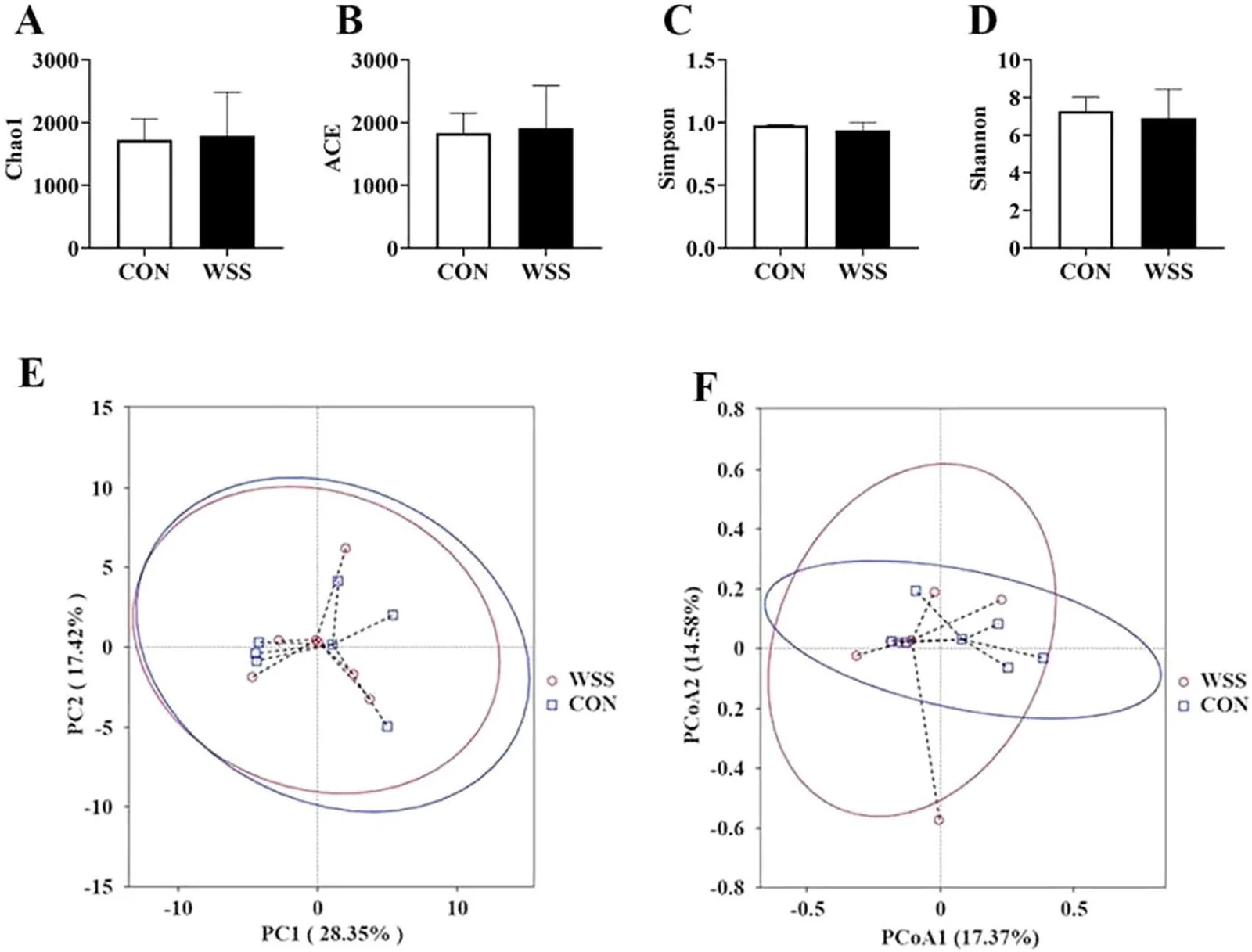
Alpha- and beta-diversity summaries of cecal microbiota in finishing pigs fed the CON or WSS diet (one pig sampled from each of six independent pens per treatment; *n* = 6 pigs per treatment). **(A)** Chao1. **(B)** Abundance-based coverage estimator (ACE). **(C)** Simpson. **(D)** Shannon. **(E)** Descriptive principal component analysis (PCA). **(F)** Principal coordinates analysis (PCoA) based on unweighted UniFrac distances. CON denotes the corn-based control diet, and WSS denotes the complete diet containing 20% whole sweet sorghum silage on an as-fed basis. Bars in panel **(A–D)** are means ± standard error of the mean (SEM). Overall community structure was tested with permutational multivariate analysis of variance (PERMANOVA).

At the phylum level, the dominant bacterial taxa in the cecal digesta were Firmicutes, Bacteroidota, Proteobacteria, Actinobacteriota, and Spirochaetota (Figure 4A). At the genus level, the displayed profiles included *Bacteroides*, *Escherichia-Shigella*, *Streptococcus*, and *Clostridium* sensu stricto, with a substantial fraction grouped as Others (Figure 4B). Exploratory linear discriminant analysis effect size (LEfSe) identified 17 taxa with differential relative abundance between treatments at an absolute linear discriminant analysis (LDA) score threshold of > 3.5 (Figures 5A, B).

**FIGURE 4:**
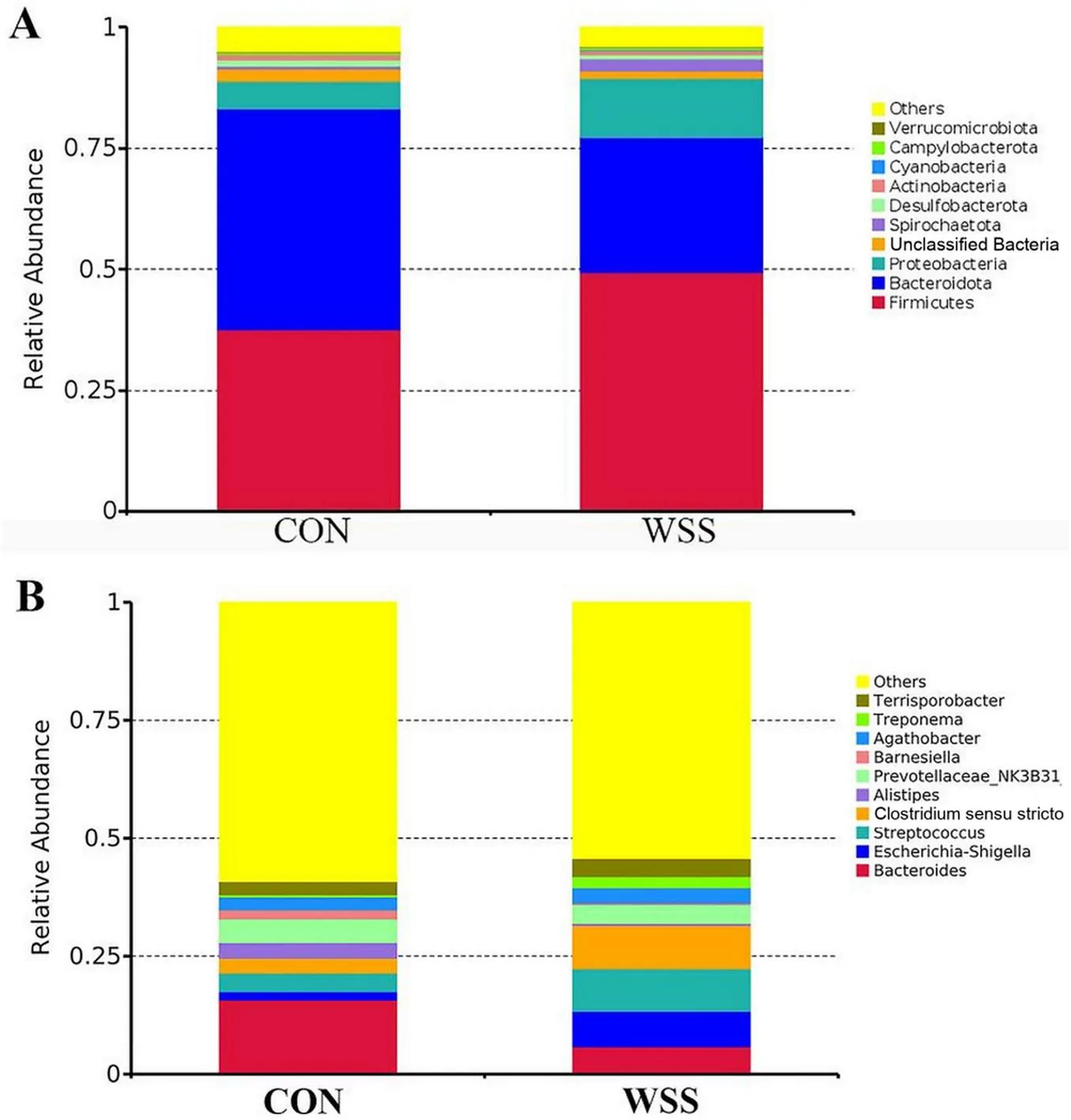
Mean relative abundance of cecal microbial taxa in finishing pigs fed the CON or WSS diet (one pig sampled from each of six independent pens per treatment; *n* = 6 pigs per treatment). **(A)** Phylum level. **(B)** Genus level. For each taxon, relative abundance was first calculated within each animal; the stacked bars represent the mean relative abundance across the six sampled animals within each treatment. Database labels were standardized for presentation: Unclassified Bacteria denotes reads not classified at the displayed rank, and *Clostridium* sensu stricto replaces a truncated database label. CON denotes the corn-based control diet, and WSS denotes the complete diet containing 20% whole sweet sorghum silage on an as-fed basis. The Actinobacteria label in panel **(A)** corresponds to Actinobacteriota in the text. Relative-abundance data are compositional.

**FIGURE 5:**
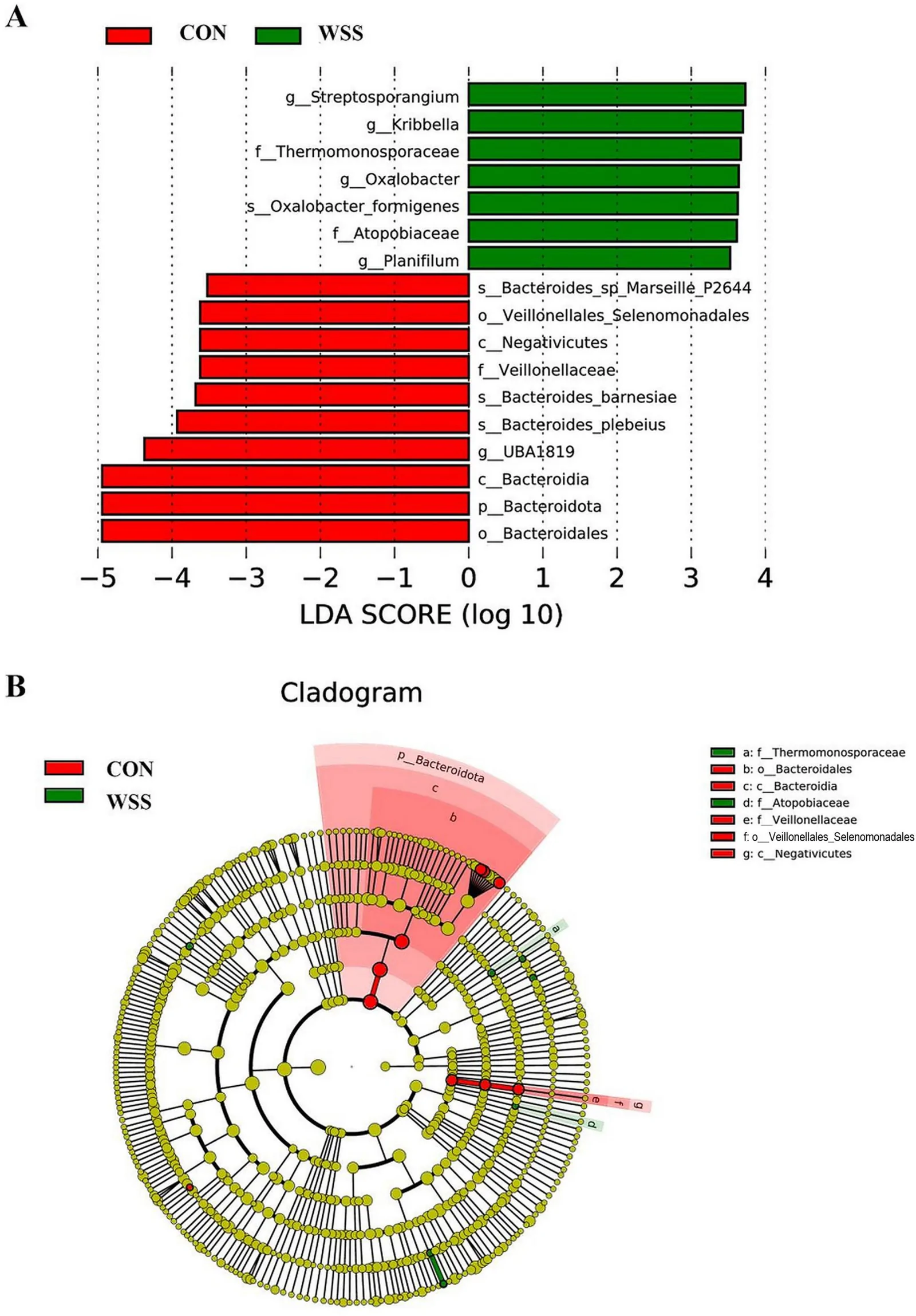
Exploratory linear discriminant analysis effect size (LEfSe) of cecal microbiota in finishing pigs fed the CON or WSS diet (one pig sampled from each of six independent pens per treatment; *n* = 6 pigs per treatment). **(A)** Taxa meeting the Wilcoxon rank-sum significance criterion of *p* < 0.05 and an absolute linear discriminant analysis (LDA) score threshold of > 3.5. **(B)** Cladogram showing their phylogenetic distribution. The panel **(B)** letter mapping is: a, f__Thermomonosporaceae; b, o__Bacteroidales; c, c__Bacteroidia; d, f__Atopobiaceae; e, f__Veillonellaceae; f, o__Veillonellales_Selenomonadales; and g, c__Negativicutes. Prefixes p_, c_, o_, f_, g_, and s_ denote phylum, class, order, family, genus, and species, respectively. CON denotes the corn-based control diet, and WSS denotes the complete diet containing 20% whole sweet sorghum silage on an as-fed basis. The analysis used compositional relative-abundance data without multiplicity adjustment and is hypothesis-generating.

## 4 Discussion

### 4.1 Growth performance

Previous studies have shown that the inclusion of whole-plant silages or other fiber-rich ingredients can modify nutrient utilization and growth performance in pigs, with the magnitude of these responses depending on inclusion level, dietary composition, and animal characteristics (Ma et al., 2024; Galassi et al., 2017; Yin et al., 2024; Pu et al., 2022). Consistent with these observations, pigs fed the WSS diet in the present study had lower ADG and ADFI and a higher F/G than those fed the CON diet. In our previous dose-response study, the highest inclusion level of whole-plant corn silage reduced final body weight and ADG and increased F/G, whereas lower inclusion levels produced less pronounced effects (Ma et al., 2024). Similarly, whole-plant corn silage impaired growth performance in heavy pigs (Galassi et al., 2017). In contrast, a 10% inclusion improved feed efficiency in Hezuo pigs, whereas other inclusion levels produced different responses (Yin et al., 2024). Collectively, these findings indicate that the effects of whole-plant silage on pig performance are not uniform but likely depend on silage type and inclusion level, as well as diet formulation, genotype, growth stage, and adaptation to dietary fiber.

In the present study, WSS replaced 20 percentage units of corn on an as-fed basis. Consequently, the WSS diet contained more moisture and crude fiber but less calculated digestible energy (DE) and CP than the CON diet. Greater dietary bulk and gastrointestinal fill, combined with lower nutrient density and the observed reduction in ADFI, may have limited daily energy and nutrient supply (Li et al., 2021). Sorghum can also contain genotype-dependent phenolic compounds, including condensed tannins that may affect nutrient use (Xu et al., 2021); however, these compounds were not quantified in the WSS used here. More importantly, the diets were not balanced for net energy or standardized ileal digestible amino acids. The growth response therefore reflects the complete dietary contrast rather than a specific effect of WSS, and nutrient-matched dose-response studies are required to define an appropriate inclusion level.

### 4.2 Carcass traits and muscle composition

Carcass traits are important determinants of carcass composition and commercial value (Lozada-Soto et al., 2022; Han et al., 2020). Compared with CON, WSS decreased backfat thickness and increased loin-eye area and marbling score. The effects of dietary fiber on carcass traits have varied among studies (Galassi et al., 2017; Yin et al., 2024; Han et al., 2020), likely because fiber source, inclusion level, energy supply, genotype, and slaughter weight alter the balance between growth and tissue deposition. The lower ADFI and lower calculated dietary DE may partly explain the reduced backfat thickness through a lower energy supply for subcutaneous lipid accretion. However, the present data do not resolve tissue-specific energy partitioning. The concurrent increase in marbling score indicates that subcutaneous and intramuscular fat indicators did not respond in parallel, but it does not demonstrate preferential intramuscular adipogenesis. Direct measurements of lipid deposition and muscle adipogenic pathways are needed to test this possibility.

The lower EE concentration in the longissimus dorsi accompanied the reduction in backfat thickness, whereas the CP concentration increased in the psoas major. Differences in energy and digestible amino acid supply, voluntary feed intake, growth rate, fiber-associated nutrient utilization, and muscle-specific metabolism could all have contributed to this pattern (Han et al., 2020; Xu et al., 2022a; Wood et al., 2008). Because whole-body lipid and protein deposition, nutrient digestibility, lipogenic activity, and muscle protein turnover were not assessed, the present findings demonstrate altered carcass and muscle composition but do not establish a specific nutrient-partitioning mechanism.

### 4.3 Meat quality and muscle chemical composition

In this trial, although most meat-quality traits were unchanged, several responses were muscle specific. In the longissimus dorsi, WSS increased pH and redness at 24 h and reduced drip loss at 48 h; in the psoas major, it reduced yellowness at 45 min and drip loss at 48 h. Postmortem pH affects protein denaturation, meat color, and water-holding capacity (Zhang et al., 2020; Suman and Joseph, 2013; Huff-Lonergan and Lonergan, 2005; Choe et al., 2008). Thus, the higher 24-h pH in the longissimus dorsi may have contributed to its greater redness and lower drip loss. This explanation does not account for the lower drip loss in the psoas major, in which pH was unchanged. Muscle glycogen, lactate accumulation, pH-decline kinetics, protein denaturation, and water distribution were not measured, so the basis of these muscle-specific responses remains unresolved (Zhang et al., 2020; Kayan and Koomkrong, 2022).

Marbling score was greater in the WSS group even though the EE concentration of the longissimus dorsi was lower, which differs from the usual positive association between marbling and intramuscular lipid (Xu et al., 2022a; Wood et al., 2008). This apparent discrepancy may reflect the different properties captured by the two measurements: visual scoring evaluates the distribution and appearance of fat flecks on the cut surface, whereas solvent extraction quantifies total extractable lipid in the sampled tissue (Wood et al., 2008; National Pork Producers Council, 1999). Spatial heterogeneity of intramuscular fat, sampling location, cut-surface characteristics, and evaluator variability may therefore allow the marbling score and chemically determined lipid concentration to change in different directions.

Beyond technological traits, WSS increased IMP concentrations and the concentrations of glutamate, aspartic acid, and tyrosine measured after total hydrolysis in both muscles, and increased phenylalanine in the psoas major. These findings demonstrate changes in muscle chemical composition. The amino acid analysis characterized the total hydrolyzed amino acid pool rather than the free amino acid pool; consequently, higher concentrations of these amino acids cannot be interpreted as greater availability of free taste-active compounds. Similarly, the higher IMP concentration is a biochemical observation and does not by itself demonstrate enhanced umami intensity or improved sensory flavor. Because free amino acids and sensory responses were not measured, the present results support an interpretation in terms of muscle chemical composition rather than sensory improvement.

The concurrent changes in jejunal morphology, cecal bacterial profiles, and muscle composition raise the possibility that intestinal responses contributed to the muscle phenotype. Recent pig studies that combined microbial, metabolite, muscle, and correlation analyses have provided evidence for diet–gut–muscle associations (Liu et al., 2026; Niu et al., 2023; Xu et al., 2023). In the present study, however, amino acid digestibility, intestinal transport, microbial metabolites, muscle metabolic pathways, and cross-trait correlations were not evaluated. The gut–muscle interpretation should therefore be regarded as a biologically plausible hypothesis rather than a demonstrated pathway.

### 4.4 Jejunal morphology

The jejunum is a principal site of nutrient digestion and absorption, and its mucosal architecture is responsive to dietary composition. WSS increased villus height and VH/CD without changing crypt depth, indicating jejunal morphological adaptation to the dietary contrast. In our whole-plant corn silage study, low and medium inclusion levels supported greater villus height and VH/CD than the highest inclusion, which was accompanied by poorer nutrient availability (Ma et al., 2024). Studies using other fiber sources likewise show that intestinal responses depend on fiber type, dose, and pig genotype (Liu et al., 2022; Pluske et al., 1996). However, a morphological response in the present study cannot be assumed to have the same functional implications as responses observed under other dietary or physiological conditions.

The increased villus height and VH/CD describe a structural response of the jejunal mucosa, rather than direct evidence of improved intestinal function. Nutrient transport, digestive enzyme activity, intestinal permeability, and barrier-related proteins were not measured; therefore, these findings do not establish enhanced nutrient absorption, digestion, or barrier integrity. The coexistence of these morphological changes with lower ADG and poorer feed efficiency also cautions against interpreting taller villi as a general improvement in intestinal function. Studies combining histomorphometry with direct functional measurements are needed to determine the physiological significance of this response.

### 4.5 Cecal microbiota

Dietary fiber escaping enzymatic digestion provides substrates for hindgut fermentation and can alter microbial community composition (Li et al., 2021; Jha and Berrocoso, 2015; Liao et al., 2024). Despite the substantial dietary contrast in the present study, no significant differences were detected in bacterial richness or diversity indices or in community structure assessed by unweighted UniFrac. Firmicutes, Bacteroidota, and Proteobacteria predominated in both groups, consistent with their common representation in the porcine gastrointestinal tract (Liao et al., 2024). The absence of a detected community-level difference is an important counterpart to the exploratory taxon-level findings: the dietary response may involve selected bacterial features without a readily detectable change in these broader community summaries. However, nonsignificant tests do not establish equivalence between diets, particularly with six sampled pigs per treatment. Moreover, sampling only at the end of the trial cannot demonstrate temporal stability or recovery after perturbation, so microbial resilience remains a hypothesis rather than an established outcome.

Within this context, exploratory LEfSe identified treatment-associated relative-abundance signals. WSS-associated features comprised *Streptosporangium*, *Kribbella*, *Oxalobacter*, an *Oxalobacter formigenes*-assigned feature, Thermomonosporaceae, Atopobiaceae, and *Planifilum*, whereas CON-associated features were concentrated within Bacteroidota/Bacteroidales, Bacteroidia, Negativicutes, Veillonellaceae/Veillonellales–Selenomonadales, *Bacteroides* lineages, and UBA1819. These findings are hypothesis-generating and indicate selected taxonomic signals rather than a broad community shift. Alpha-diversity indices summarize within-sample richness and diversity, whereas unweighted UniFrac evaluates phylogenetic community membership rather than relative-abundance changes. Thus, selected relative-abundance differences can occur without a significant difference in these broader community summaries.

Biological interpretation should focus on plausible hypotheses rather than assign a specific function to every differential taxon. The *Oxalobacter formigenes*-assigned feature provides one such hypothesis because oxalate degradation has been described for this organism in other mammalian settings (Daniel et al., 2021). Nevertheless, the present 16S rRNA gene annotation does not establish species identity or metabolic activity, and neither dietary oxalate nor intestinal oxalate turnover was measured. Its functional relevance in the porcine cecum therefore remains uncertain. For *Streptosporangium*, *Kribbella*, and *Planifilum*, the present data do not establish an active contribution to fiber utilization in these pigs; detailed extrapolation from environmental isolates is therefore unwarranted. The detected sequences also cannot distinguish resident organisms from transient or feed-associated microbial material. None of these relative-abundance signals establishes a beneficial, harmful, or pathogenic effect.

Other silage-based pig studies have linked diet-associated microbial shifts to meat-quality traits using microbial, metabolite, or correlation analyses (Niu et al., 2023; Xu et al., 2023), and recent multi-omic work supports a broader diet–gut–muscle framework (Liu et al., 2026). These studies provide a rationale for investigating microbial contributions to the concurrent jejunal and muscle responses observed here, but such contributions were not tested in the present experiment. However, cecal pH, short-chain fatty acids, absolute abundance, microbial genes, metabolic activity, and microbiota–phenotype correlations were not measured. The LEfSe signals are also exploratory relative-abundance features without multiplicity adjustment. Targeted quantitative polymerase chain reaction, absolute quantification, metabolomics, and metagenomic or metatranscriptomic analyses are needed to determine whether these taxa or their metabolites contribute to intestinal or muscle phenotypes.

Finally, the two diets were not matched for DM, available energy, CP, or digestible amino acids, and each treatment included six pens. Slaughter pigs were selected by proximity to their pen mean final BW within a designated sex rather than by unrestricted random sampling. Consequently, the slaughter-based outcomes describe these selected pigs and may not capture the full within-pen variation; potential selection bias cannot be excluded. Multiple phenotypic and taxonomic outcomes were evaluated, while digestibility, sensory responses, and economic outcomes were not assessed. The findings therefore describe responses to the complete dietary contrast and identify testable mechanisms, rather than establishing an ingredient-specific or microbiota-mediated causal pathway.

## 5 Conclusion

In summary, as outlined in the graphical summary (Figure 6), replacing 20 percentage units of dietary corn with WSS on an as-fed basis reduced growth performance while producing concurrent changes in selected carcass and meat-quality traits in heavy finishing pigs. WSS reduced ADG and ADFI and increased F/G, while decreasing backfat thickness and 48-h drip loss and increasing loin-eye area, marbling score, and IMP concentrations. The increases in IMP and selected total hydrolyzed amino acids indicate changes in muscle chemical composition and do not establish improved flavor or umami intensity. Jejunal villus height and VH/CD increased without a significant change in crypt depth. These changes indicate jejunal morphological adaptation and do not establish improved intestinal function. At the microbial level, no significant differences were detected in alpha-diversity indices or unweighted UniFrac community structure between diets, although exploratory LEfSe identified selected treatment-associated taxa.

**FIGURE 6:**
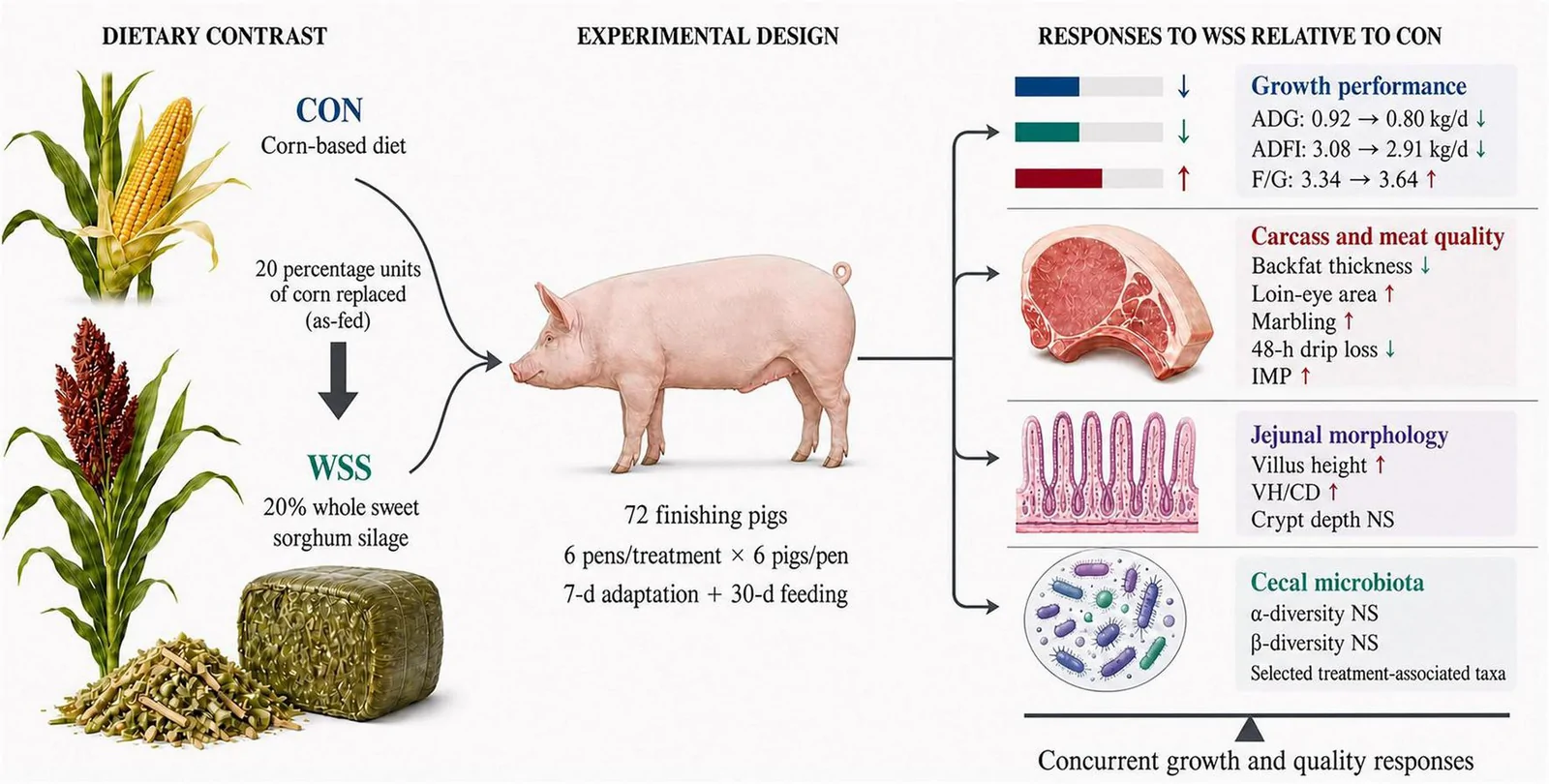
Graphical summary of the experimental design and measured responses to partial replacement of corn with whole sweet sorghum silage (WSS) in heavy finishing pigs. NS denotes no significant difference detected. The concurrent intestinal, microbial, carcass, and meat-quality responses are associative and do not establish a causal gut-muscle mechanism.

These concurrent responses show that the WSS-containing dietary contrast was associated with production traits, intestinal morphology, and selected cecal bacterial features, but they do not establish a causal gut–muscle pathway. The dietary treatments were not matched for dry matter, available energy, crude protein, or digestible amino acids, and microbial metabolites, barrier function, nutrient transport, muscle metabolic pathways, and cross-trait correlations were not measured. The proposed mechanistic links therefore remain hypothetical. Nutrient-balanced dose-response studies integrating microbial function, intestinal physiology, and muscle metabolism are needed before an optimal practical inclusion level for WSS can be recommended.

## Data Availability

The raw data supporting the conclusions of this article will be made available by the authors, without undue reservation.

## Appendix

**TABLE A1:** Per-sample sequencing yield and quality-control summary.

Sample ID	Raw PE (pairs)	Merged tags	Quality-filtered tags	Non-chimeric tags	Analysis depth
Control1	82,719	81,865	81,095	61,353	41,476
Control2	83,346	82,527	81,906	63,008	41,476
Control3	85,429	83,289	82,692	64,224	41,476
Control4	86,065	84,718	84,223	63,247	41,476
Control5	72,678	71,395	70,949	50,278	41,476
Control6	80,624	79,126	78,581	61,118	41,476
Treatment1	69,914	69,253	68,745	50,317	41,476
Treatment2	86,385	85,146	84,682	61,745	41,476
Treatment3	82,112	80,606	80,158	59,821	41,476
Treatment4	82,452	81,306	80,740	63,847	41,476
Treatment5	74,855	74,150	73,692	55,975	41,476
Treatment6	81,856	80,212	79,394	62,681	41,476

Sample identifiers are reproduced as recorded in the original sequencing report. Raw PE denotes raw paired-end read pairs. Merged tags are assembled paired-end sequences; quality-filtered tags are retained after quality filtering; non-chimeric tags are the effective sequences remaining after chimera removal. The OTU table was normalized to 41,476 sequences per sample for alpha-diversity and UniFrac analyses.
